# Biomedical Applications of Thermosensitive Hydrogels for Controlled/Modulated Piroxicam Delivery

**DOI:** 10.3390/gels9010070

**Published:** 2023-01-15

**Authors:** Snežana Ilić-Stojanović, Ljubiša Nikolić, Vesna Nikolić, Ivan Ristić, Suzana Cakić, Slobodan D. Petrović

**Affiliations:** 1 Faculty of Technology, University of Niš, Bulevar Oslobodjenja 124, 16000 Leskovac, Serbia; 2 Faculty of Technology, University of Novi Sad, Bulevar Cara Lazara 1, 21000 Novi Sad, Serbia; 3 Faculty of Technology and Metallurgy, University of Belgrade, 11000 Belgrade, Serbia

**Keywords:** hydrogels, *N*-isopropylacrylamide, 2-hydroxypropyl methacrylate, DSC, drug carrier, modulated drug release, piroxicam

## Abstract

The objectives of this study are the synthesis of thermosensitive poly(*N*-isopropylacrylamide-*co*-2-hydroxypropyl methacrylate), p(NiPAm-HPMet), hydrogels and the analysis of a drug-delivery system based on piroxicam, as a model drug, and synthesized hydrogels. A high pressure liquid chromatography method has been used in order to determine both qualitative and quantitative amounts of unreacted monomers and crosslinkers from polymerized hydrogels. Swelling kinetics and the order of a swelling process of the hydrogels have been analyzed at 10 and 40 °C. The copolymers’ thermal properties have been monitored by the differential scanning calorimetry (DSC) method. DSC termograms have shown that melting occurs in two temperature intervals (142.36–150.72 °C and 153.14–156.49 °C). A matrix system with incorporated piroxicam has been analyzed by using FTIR and SEM methods. Structural analysis has demonstrated that intermolecular non-covalent interactions have been built between side-groups of copolymer and loaded piroxicam. Morphology of p(NiPAm-HPMet) after drug incorporation indicates the piroxicam presence into the copolymer pores. Kinetic parameters of the piroxicam release from hydrogels at 37 °C and pH 7.4 indicate that the fluid transport mechanism corresponds to Fickian diffusion. As a result, formulation of thermosensitive p(NiPAm-HPMet) hydrogels with incorporated piroxicam could be of interest for further testing as a drug carrier for modulated and prolonged release, especially for topical administration.

## 1. Introduction

The first representative of the non-steroidal anti-inflammatory drugs (NSAID) in the oxicam group class (derivatives of 4-OH-1,2-benzothiazine carboxamide) is 4-hydroxy-2-methyl-N-pyrid-2-yl-2H-1,2-benzothiazine-3-carboxamide-1,1-dioxide, or piroxicam. It shows polymorphism, and can exist in several prototropic forms [1] and conformations [2]. In its basic state, piroxicam exists both as closed and an open conformation. Conformations are more favorable in non-polar solvents, as a result of intramolecular proton transfer from the hydroxyl group’s benzothiazine ring to the ortho-carbonyl group. The enol form of the hydroxyl group and amide carbonyl oxygen is converted into the keto-tautomeric form [2,3,4]. In its basic state, piroxicam is found in a neutral form, but it also exists as a zwitter ion. Spectroscopically, it is difficult to prove this behavior of piroxicams, so both neutral and zwitterionic forms are collectively called “globally neutral” [5]. Intramolecular proton transfer by piroxicam depends on the polarity of the solvent and the possibility of forming hydrogen bonds [6].

Piroxicam is an analgesic, antipyretic and anti-inflammatory drug, which is very effective in various arthritis and postoperative conditions. It reduces the number of polymorphnuclear cells in the joint fluid, thereby exerting an additional anti-inflammatory effect. Resorption of piroxicam from the GIT is rapid. The maximum concentration in blood occurs between 3 to 5 h after administration, and the binding to plasma proteins is 99% effective. The elimination half-time of piroxicam is about 50 h and allows single dosing and a long duration of the therapeutic effect. Its use can lead to gastrointestinal poisoning, tinnitus, dizziness, headache, rash and itching. The most serious side effects when orally administered are peptic ulceration, gastrointestinal bleeding and hepatotoxicity [7]. It may also cause skin sensitivity to sunlight. Although the most reactive photoactive groups of 2-arylpropionic acid are not included, it has specific characteristics that make it attractive for photochemical studies [6]. Sasaki and colleagues investigated in vivo the photosensitivity of ampiroxicam, which is obtained by biotransformation of piroxicam, and showed that it is more sensitive to UV A radiation [8]. Kurumaji also demonstrated photosensitivity to ampiroxicams [9]. The photostability of piroxicam was improved in the inclusion complex 2-hydroxypropyl-β-cyclodextrin: piroxicam for a period of up to 30 days [10].

Despite many properties that make it suitable for medical use, piroxicam is hydrophobic and has a low solubility in water. In order to overcome the problem of poor solubility, numerous studies by scientists were carried out in order to obtain an effective formulation for better bioavailability. The company SS Pharmaceutical Co patented piroxicam tablets based on an ethanolic solution of hydroxypropylcellulose and lactose and the preparation process [11]. Formulations of piroxicam with β-lactose and poly(vinylpyrrolidone) K12 and K90 shows improved solubility [12]. Microspheres based on pectin as a carrier for piroxicam were tested in vitro and in vivo, and proved to be suitable for ophthalmological application [13]. Piroxicam orodispersible tablets are solid dispersions with microcrystalline cellulose and dextrose, formulated to improve taste [14].

Acid base equilibrium leads to the existence of different prototropic forms, which have been used to study organized structures such as micelles or cyclodextrins [15,16]. The inclusion complex of β-cyclodextrin (β-CD) and piroxicam was investigated by the X-ray diffraction method [17]. The piroxicam release from the complex with β-CD varies depending on the preparation procedure [18]. Scanning electron microscopy (SEM) analysis has shown the morphological differences of piroxicam, the physical mixture and the complex of piroxicam with β-cyclodextrin. The ratio of free and bound piroxicam in inclusion complexes was determined [19]. The inclusion complexes of piroxicam and 2-hydroxypropyl-β-cyclodextrinshowed significant improvement in solubility. A ^13^C NMR analysis indicates the possible incorporation of piroxicam into 2-hydroxypropyl-β-cyclodextrin via the pyridine ring [20].

Formulations with controlled release of piroxicam in chronic pain are particularly suitable for improving the effectiveness of treatment [21]. The copolymer poly(oxyethylene)-poly(oxypropylene)-poly(oxyethylene), better known as poloxamer, is a potential basis for the production of thermosensitive hydrogels [22,23]. Thus, piroxicam formulations with poloxamers were prepared. The use of such formulations in clinical practice has been shown to be beneficial, as the concentration of piroxicam in plasma is maintained for 4 days, which enables sustained release and better bioavailability [24]. Novel intra-articularly injectable thermosensitive hydrogel for sustained piroxicam delivery, which is based on the physical mixing of Pluronic F-127 and hyaluronic acid, was developed by Jung et al. [25]. Soni and colleagues studied the potential application of chitosan and hydroxypropyl methyl cellulose matrices for specificsustained release of piroxicam in the stomach by using gastroretention technology [26]. Anti-inflammatory biological agents from piroxicam, which are incorporated into poly-ε-caprolactone nano-particles, were prepared and characterized for sustained NSAID delivery for a period of 12 days [27]. Poly-[(3-acrylamidopropyl)-trimethylammoniumchloide-b-*N*-isopropyl acrylamide] hydrogels encapsulated with calcium alginate microspheres were used to deliver hydrophobic piroxicam as a model drug [28]. The nanoparticles based on Eudragit S100 with incorporated piroxicam were obtained by nanoprecipitation technique and dispersed in a Carbopol 934 hydrogel and assessed for transdermal administration. Piroxicam release from these pH-sensitive nanocarriers and the hydrogel show significant transdermal increase via mice [29]. Topical formulations of piroxicam are produced in form of a gel, and a semisolid dosage form and they posses high efficacy and ease of application. Boonme and colleagues investigated microemulsion formation of various non-ionic systems for incorporation of 0.5% w/w piroxicam [30]. They have developed the hyaluronic acid: piroxicam formulation in a ratio of 1:1 with a synergistic effect and showed greater effect than either one of them individually [31].

The aims of this investigation are biomedical application, characterization and analysis of drug delivery system based on piroxicam as a model drug with poly(*N*-isopropylacrylamide-*co*-2-hydroxypropyl methacrylate), p(NiPAm-HPMet) hydrogels. The profile of piroxicam release from this matrix system with synthesized thermosensitive hydrogels was studied at physiological body temperature and pH 7.4. Potential administrations of similar hydrogels for controlled delivery of some NSAIDs, e.g., caffeine [32], phenacetin [33], ibuprofen [34], and naproxen [35] were presented in previous studies. No equivalent study about matrix system for controlled piroxicam delivery has been found in the accessible published works. Applied synthesized smart hydrogels manifest phase transitions when they swell in response to temperature changes and make these materials convenient, with superior properties to the other previously analyzed hydrogels. For this reason, they are interesting as matrix systems for piroxicam delivery in patient’s body temperature. Remarked thermosensitive properties of the p(NiPAm-HPMet) hydrogels designated that they could be a good candidate for future study as the carrier for controlled/modulated piroxicam delivery, especially for prolonged topical administration.

## 2. Results and Discussion

### 2.1. Hydrogel Polymerization

The polymerization of p(NiPAm-HPMet) hydrogels was realized using comonomer 2-hydroxypropyl methacrylate (HPMet) (15 mol%) and ethylene glycol dimethacylate (EGDM) (0.5, 1.0, 1.5, 2.0 and 3.0 mol%) as crosslinkers. The sample with 0.5 mol% of crosslinker EGDM was not used for further analysis because of stays in a liquid state after synthesis without the need for hydrogel consistency. After the purification from unwanted and unreacted products, polymerized hydrogels in the xerogel stage were analyzed.

### 2.2. Residual Reactants Analysis

The high pressure liquid chromatography (HPLC) is a very useful technique for detection qualitative and quantitative amount of the unreacted residual reactants (NiPAm and HPMet as monomers and EGDM as crosslinker) after synthesis in the polymerized hydrogels. Production of polymers with a minimum amount of unreacted reactants is a necessary prerequisite for their applications because of their toxicity in much higher amounts, especially for biomedical applications. We recorded a retention time (R_t_) of 2.597 min corresponding to the monomer *N*-isopropylacrylamide (NiPAm); an R_t_ of 2.316 min corresponding to the HPMet; and an R_t_ of 2.817 min corresponding to the EGDM. The wavelength value at which the maximum absorption in the UV region, λ_max_, occurs for NiPAm is 225 nm; for HPMet, it is 215 nm; and for EGDM, it is 205 nm. The detection wavelength of 220 nm was chosen because the methanol samples were analyzed for all three reactants: NiPAm, HPMet and EGDM. The calculated amount of residual, unreacted reactants after the polymerization, in relation to the initial amount in the reaction mixture in mass% is presented in Table 1.

Based on the presented results, it is obvious that the achieved conversion from the comonomers and crosslinker to the polymer was satisfied. Obtained amounts of residual reactants were within tolerance limits for all polymerized p(NiPAm-HPMet) samples [36,37]. The International Standard ISO 1567:1999, Dentistry—Denture base polymers, defined the tolerance limit for the residual methyl methacrylate maximums: 2.2% for hot, and 4.5% for cold polymerized product. In dental products, the tolerance limits for unreacted methyl methacrylate are from 1% to 3% [38]. The content of residual monomers from 0.5% to 1.0% or more is frequently found in industrial polymers products [39].

### 2.3. Differential Scanning Calorimetry (DSC) Analysis

**Figure 1:**
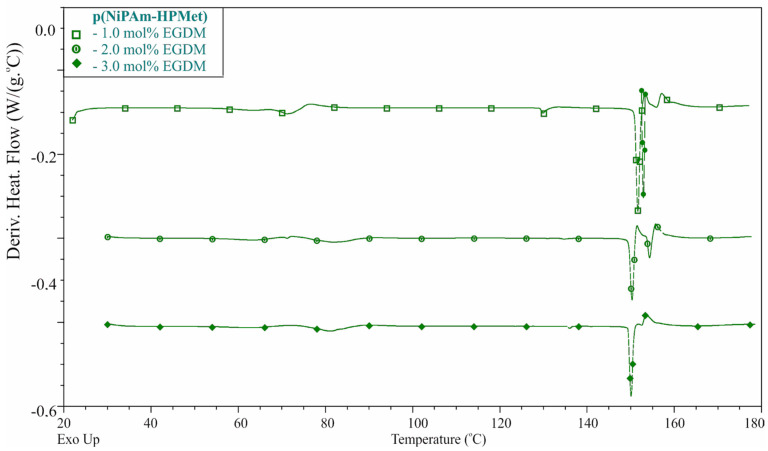
Differential scanning calorimetry (DSC) curves (first derivative of heat flow) of the synthesized poly(*N*-isopropylacrylamide-*co*-2-hydroxypropyl methacrylate) xerogels.

The phase transitions identification of p(NiPAm-HPMet) copolymers with 15 mol% of 2-hydroxypropyl methacrylate, and with 1.0, 2.0 or 3.0 mol% of the ethylene glycol dimethacylate in the range of 30–180 °C, are shown in Figure 1 as the first derivative of heat flow. Phase transitions are observed in a differential scanning calorimetry (DSC) thermogram with low intensity of the glass transitions and strong peaks from melting of crystalline regions of analyzed samples.

Values of the temperatures of glass transition (*T*_g_), melting process (*T*_m_), and the melting enthalpy for the investigated p(NiPAm-HPMet) xerogels are shown in Table 2.

The glass transition temperature presents the process where the hydrogel sample is heated and changes from a glassy state to a soft state (“viscous liquid” but not melted). This is characteristic temperature range for amorphous regions, and it depends on the chemical structure. The DSC thermograms of synthesized p(NiPAm-HPMet) samples indicate the glass transition in more than one temperature interval. The xerogel with 1.0 mol% of crosslinker shows two glass transitions temperatures (at 71.26 °C and 129.81 °C), while xerogels with 2.0 and 3.0 mol% of EGDM show three glass transition temperatures (at about 63 °C, 81 °C and 135 °C). The absence of a glass transition peaks from p(HPMet), which occurs at 95 °C [40], confirmed that its crosslinking with p(NiPAm) was successful [41]. Little variations in the glass transition temperatures of copolymer hydrogels, which were proportional to the molar amountsof the crosslinker, were detected. The increase of T_g3_ of p(NiPAm-HPMet) with the higher content of EGDM is probably because of crosslinking density increase. This is reasonable because the length of the chain between crosslink points is reduced, which results in the increase in the glass transition temperature.

The melting temperature characterizes the process during heating when the crystalline regions of hydrogels change physical state and become a disordered viscous liquid phase. Polymer chains with different lengths, branching and crosslinks have different times to melt, because of they need various energies and they melt in the broader melting temperature. Analysis of presented data in DSC thermogram for the samples of p(NiPAm-HPMet) with 1.0, 2.0 and 3.0 mol% of EGDM exhibits that melting occurs in two temperature intervals (first from 150.72 °C to 142.36 °C and second from 153.14 °C to 156.49 °C). The melting temperature slightly decreases with the increase of the crosslinking degree, whereby the intensity of the second peak and the melting enthalpy decreases. The melting enthalpy also decreases with increase of the crosslinking degree with higher EGDM content, and it has the values in the range 2.94–6.48 J∙g^−1^. This is expected, since the increase of crosslinking diminishes the number of unconnected polymer chains and forms crystalline regions. The obtained results confirmed structural irregularity, good compatibility and miscibility of NiPAm and HPMet comonomers [39,41]. They are semi-crystalline, their warming leads to melting, and they are thermally stable up to temperatures of about 150 °C. This allows for their sterilization, which is very important for the biomedical usage of p(NiPAm-HPMet) hydrogels.

### 2.4. Swelling Study

The analysis of p(NiPAm-HPMet) hydrogels in the fluid with pH value 7.4, at 10 °C and 40 °C, was performed for a better understanding of the swelling kinetics mechanism, especially for the decision on the best conditions for application as a carrier for piroxicam incorporation [42,43]. In previously published study [35], naproxen was loaded at 5 °C, but for slightly hydrophobic piroxicam, a slightly higher temperature (at 10 °C) provides better loading.

#### 2.4.1. Swelling Reversibility

**Figure 2:**
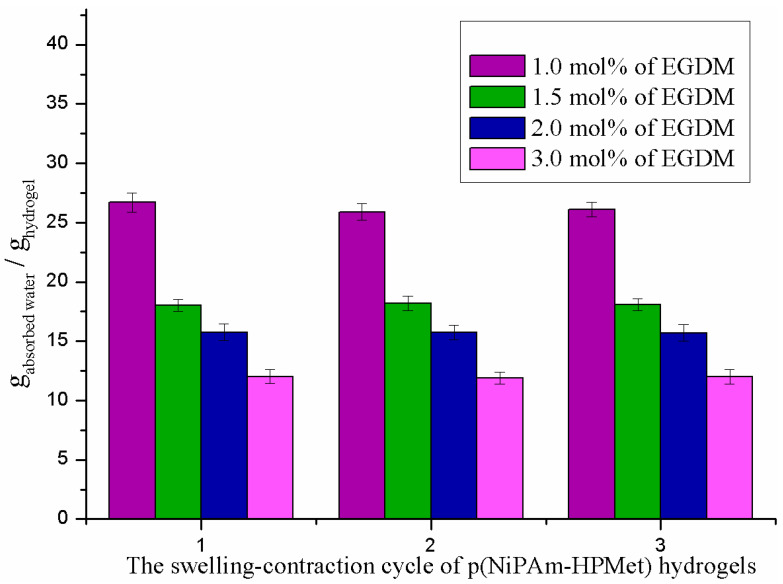
Swelling reversibility of p(NiPAm-HPMet) hydrogel at alternating swelling/contraction for three cycles under the temperature change from 10 °C to 50 °C. Error bars represent the standard deviation of the means of three replicates.

Testing of the swelling reversibility of synthesized p(NiPAm-HPMet) samples was supervised in three cycles. Swelling reversibility was analyzed with the equilibrium swelling degree (Equation (4)). Weighted xerogels were swollen to the equilibrium state at 10 °C, and after that heated to 50 °C in order to notice their contraction due to desorption. The obtained results for the maximum water absorption of p(NiPAm-HPMet) hydrogel samples that pass through the alternating swelling/contraction cycles, in dependance of temperature change from 10 to 50 °C are shown in Figure 2. All samples of p(NiPAm-HPMet) hydrogel have a high water absorption capacity in the first cycle (26.70–12.03 g_water_/g_hydrogel_). In the second and third swelling/contraction cycles, their ability to absorb water remains the same, and confirms that hydrogels show good reproducibility. The reversibility of the swelling/contraction process depending on temperature is based on the establishment of an equilibrium between hydrophilic and hydrophobic groups in the polymer. This equilibrium is responsible for the formation and breaking of intermolecular hydrogen bonds. When the temperature rises from 30 °C to 38 °C, at the lower critical solution temperature (LCST), intermolecular hydrogen bonds break. This causes the contraction of the hydrogel and the releaseof water molecules from the hydrogel matrix, due to the formation of dominant intramolecular backbone interactions. Experimental results for reversibility of swelling with pulsating behavior agree with literature data for p(NIPAM-HPMet) hydrogels [39,44].

#### 2.4.2. Analysis of the Hydrogels Swelling Kinetics

The calculated values of equilibrium swelling ratio, *α*, diffusion exponent, *n*, kinetic constant, *k*, the correlation coefficient (R^2^) and the diffusion coefficient, D, determined using Equations (8), (12), (14) and (15) for p(NiPAm-HPMet) hydrogels in the fluid with pH value 7.4, at 10 °C and 40 °C are presented in Table 3 and Table 4, respectively.

The analysis of the swelling kinetics results at 10 °C in the fluid with pH value 7.4 (Table 3) shows that the transport mechanism into the p(NiPAm-HPMet) samples with 1.0 and 3.0 mol% of EGDM obeys Fickian diffusion, and is called “Less Fickian” diffusion. Fluid penetration is notably slower than the relaxation of the polymer chains. The fluid transport into the p(NiPAm-HPMet) hydrogels samples with 2.0 mol% of EGDM corresponds to Fickian diffusion mechanism (Case I), and the diffusion degree is lower than the polymer chain relaxation degree. The anomalous diffusion mechanism, “non-Fickian diffusion,” corresponds to the p(NiPAm-HPMet) hydrogels sample with 1.5 mol% of EGDM. It occurs when the hydrogel swelling process is controlled both by fluid diffusion into the polymer matrix and polymer chain relaxation. Similar values for the diffusion exponent of p(NiPAm-HPMet) copolymers at 25 °C have been previously published [32,35].

The mechanism of fluid transport in contracted p(NiPAm-HPMet) hydrogels at 40 °C, above the LCST (Table 4) for all samples, was subject to Fick’s law, so that the diffusion controls the process of swelling, and it is a slower process [45].The decreasing EGDM content, i.e., reducing the crosslinking degree, leads to an increase in the value of n, which means that the swelling rate increases [46,47]. In a previously published paper [35], the authors analyzed the temperature responsiveness of synthesized p(NiPAm-HPMet) hydrogels from 5 °C to 60 °C. According these results, the analyzed hydrogel samples, during heating, reached volume phase transition temperatures (VPTT) in the range of 30–38 °C [35]. After a more precise evaluation in the lesser temperature intervals (of 5 °C), the VPTT were 34.0, 34.3, 34.6 and 34.9 °C for the analyzed samples, with 1.0, 1.5, 2.0 and 3.0 mol% of EGDM, respectively, which are similar to those found by Cai and colleagues [44].

The kinetic constant, k, values are little higher at 40 °C in relation to values at 10 °C because of the free volume reduction after the contraction of the polymer chains [33,35]. The calculated values of the diffusion coefficient, *D*, at 10 °C, are in the range 8.872 × 10^−7^–1.041 × 10^−5^ cm^2^/min. At a temperature above the LCST, i.e., at 40 °C, the values of the diffusion coefficient are in the range 1.206 × 10^−3^–5.367 × 10^−5^ cm^2^/min, and indicate an increase in the values for all samples of copolymeric hydrogels, i.e., faster diffusion. The increase of the diffusion coefficient with the increase of temperature has been documented in the literature [48,49,50,51].

#### 2.4.3. Determination of the Order of Reaction for Swelling Process

The values of the experimentally obtained *α*_e_ (exp), the calculated normalized equilibrium swelling degree *α*_e_, the rate constant, *K*, and the value of the linear correlation coefficient, R^2^, for p(NiPAm-HPMet) hydrogels were determined using logarithmic Equations (19) and (20) for the first- and second-order at 10 and 40 °C are shown in Table 5 and Table 6. The condition is that the dependence 20 is linear. Experimentally obtained water content values, *α*_e_ (exp), are listed for comparison.

At the temperature of 10 °C, the experimentally determined and calculated values of the normalized equilibrium swelling degree for p(NiPAm-HPMet) hydrogels were in concordance (Table 5). The correlation coefficients values were close to 1, and indicate good agreement with the experimental results with the presumed order of reaction, but they are a better fit for the second-order reaction.

Similar results were obtained at 40 °C (Table 6). The experimentally determined and calculated values of normalized equilibrium swelling degree for p(NiPAm-HPMet) hydrogels were in concordance. The correlation coefficients values were close to 1, and indicate that it agrees well with the experimental results with the presumed order of reaction, but they are a better fit for the second-order reaction. The values of the reaction rate constants, *K*, were within the same order of magnitude for all samples (Table 5 and Table 6). It could be stated that the swelling behavior of p(NiPAm-HPMet) hydrogels shows a slightly larger deviation from the first-order reaction at both investigated temperatures (10 °C and 40 °C). As a result, it can be concluded that the swelling process of p(NiPAm-HPMet) samples follows the second-order reaction.

### 2.5. Fourier Transform Infrared Spectroscopy (FTIR) Analysis

#### 2.5.1. FTIR Spectrum Analysis of Piroxicam

**Figure 3:**
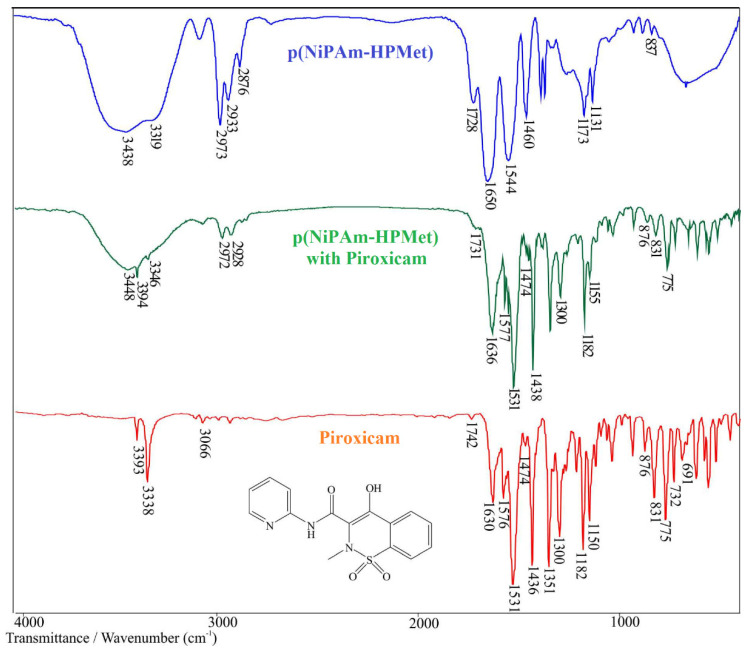
FTIR spectra of: pure p(NiPAm-HPMet) hydrogel, p(NiPAm-HPMet) hydrogel with incorporated piroxicam and piroxicam with the structural formula.

The FTIR spectrum of piroxicam, with the maximums of the characteristic absorption bands, is given in Figure 3 and Table 7. A sharp band of medium intensity, originating from the stretching vibrations of the -OH group, ν(OH), gives an absorbance maximum at 3338 cm^−1^. In this area, the band of weak intensity with a maximum at 3393 cm^−1^ corresponds to the stretching vibration of the -NH group, secondary amide, ν(NH), from the characteristic needle shape of the polymorphic form of piroxicam, which was found and discussed in the works of other authors [18,26,52]. Stretching C-H vibrations from the pyridine ring give a band with a maximum at 3066 cm^−1^. The absorption band of medium intensity with a maximum at 1630 cm^−1^ is the result of stretching vibrations, ν(C=O), of “Amide bands I” from the secondary amide, and the intense band at 1531 cm^−1^ corresponds to the bending vibrations of the N-H group, δ(NH), “Amide band II”, which is in agreement with data from the literature [53]. “Amide band III” is a band originating from C-N stretching vibrations, ν(C-N), and in the spectrum gives a band with a maximum at 1300 cm^−1^. Absorption bands originating from stretching vibrations from the conjugated benzene ring, ν(C=C), are located in the spectrum at 1560 and 1474 cm^−1^. In the FTIR spectrum of piroxicam, there is a band with a maximum at 1436 cm^−1^, which confirms the existence of in-plane bending vibrations of the -OH group, δ(OH). Compounds that have an S=O bond in their structure give bands of stretching vibrations that are very intense. These bonds are present in the structure of piroxicam, and the characteristic bands in the spectrum reach maximums at 1351 cm^−1^ and 1182 cm^−1^, and originate from asymmetric ν_as_(SO_2_) and symmetric ν_s_(SO_2_) stretching vibrations, respectively. Pyridine in the spectrum is characterized by a band of stretching vibrations of the C-N bond, ν(C-N), at 1576 cm^−1^ and two bands with maximums at 732 cm^−1^ and 691 cm^−1^, originating from out-of-plane bending vibrations ν(C-H,) typical for monosubstituted benzene. In the region 660–900 cm^−1^, bands with maximums at 876, 831 and 775 cm^−1^ are observed, which originate from in-plane bending vibrations of the aromatic nucleus δ(C-H).

#### 2.5.2. FTIR Spectrum Analysis of p(NiPAm-HPMet) with Incorporated Piroxicam

The structural changes in the p(NiPAm-HPMet) copolymer with incorporated piroxicam are shown by the FTIR spectrum in Figure 3, and the maximum shifts of the characteristic absorption bands are given in Table 7.

Analysis of the FTIR spectrum of p(NiPAM-HPMet) was presented in the previous study of authors [35] and applied for this analysis.

In the FTIR spectrum of the p(NiPAm-HPMet) copolymer with incorporated piroxicam (Figure 3), changes in the position and intensity of certain bands were observed, indicating intermolecular interactions of the free -OH group, primarily with the carbonyl C=O group. The absorption band with a maximum at 3449 cm^−1^ from the stretching vibrations of the hydroxyl group, ν(OH), in the spectrum of the copolymer with piroxicam, was shifted by 11 units to higher wave numbers compared to the spectrum of the copolymer. The position of the maximum from the stretching vibrations of -OH groups from piroxicam in the spectrum of the copolymer with piroxicam occurs at 3346 cm^−1^ and is shifted by 7 units in relation to the spectrum of piroxicam. These shifts indicate that the -OH group could participate in the construction of the intermolecular hydrogen bond.

The needle-shaped band is expanded and has a characteristic shape for polymorphic forms, as described in the literature [18,52]. The band of stretching vibrations of the -NH group of the secondary amide, ν(NH), of piroxicam does not show a shift of the maximum in the spectrum of the copolymer with piroxicam, which indicates that it did not participate in the formation of the hydrogen bond. The stretching vibration of the C=O group from the ester provides a band with an absorption maximum at 1731 cm^−1^ in the spectrum of the copolymer with piroxicam, and it is shifted relative to the position of the same band in the spectrum of the copolymer by 4 units to higher wavenumbers, and by 11 units relative to piroxicam towards lower wavenumbers. The mentioned movements may indicate the interaction of the drug with the copolymer and the formation of hydrogen bonds. The maximum of “Amide band I”, ν(C=O), in the spectrum of the copolymer with piroxicam occurs at 1636 cm^−1^ and is shifted to lower wave numbers by 14 units compared to the spectrum of the copolymer, and by 6 units to higher wave numbers in relation to the spectrum of piroxicam. The position of “Amide band II”, δ(N-H), in the spectrum of the copolymer with piroxicam appears at 1531 cm^−1^ and is shifted by 13 units to lower wavenumbers compared to the same band in the spectrum of the copolymer, which indicates that -NH from amide groups of the copolymer participated in the interaction with the drug. In the spectrum of the copolymer with piroxicam, there are bands of stretching vibrations of the SO_2_ group from piroxicam (1353 cm^−1^ of asymmetrical, ν_as_(SO_2_), and 1182 cm^−1^ of symmetrical, ν_s_(SO_2_)) (Figure 3 and Table 7) and their shifts were not observed, indicating that the SO_2_ group did not participate in the formation of hydrogen interactions. The FTIR analysis of the spectra of p(NiPAm-HPMet) copolymer, piroxicam and p(NiPAm-HPMet) copolymer with incorporated piroxicam indicates the formed interactions of the type of hydrogen bonds between the secondary -OH group from piroxicam, or copolymer, and the -NH group of the copolymer, on the one hand, and the carbonyl group of C=O piroxicam and copolymer, on the other. Interactions between p(NiPAm-HPMet) copolymer and piroxicam are of the non-covalent type, since their shiftings are small.

### 2.6. Piroxicam Incorporation Efficiency into p(NiPAm-HPMet) Hydrogels

Piroxicam, as a model drug from the NSAID group, was applied for analysis of obtained p(NiPAm-HPMet) samples as carriers for controlled/modulated delivery. The incorporated amount of piroxicam into p(NiPAm-HPMet) samples was calculated based on the weight difference before and after the swelling in the prepared piroxicam fluid. The incorporation efficiency of piroxicam for the tested p(NiPAm-HPMet) hydrogels was calculated using Equation (12) and the obtained results are given in Table 8.

The maximum of piroxicam available mass for loading into p(NIPAM-HPMet) hydrogels is in percentage from 38.45% to 66.34%. A tendency to increase the incorporation of the piroxicam into the hydrogels with a decrease in the molar ratio of EGDM, i.e., with crosslinking density of the hydrogels, was evident.

**Figure 4:**
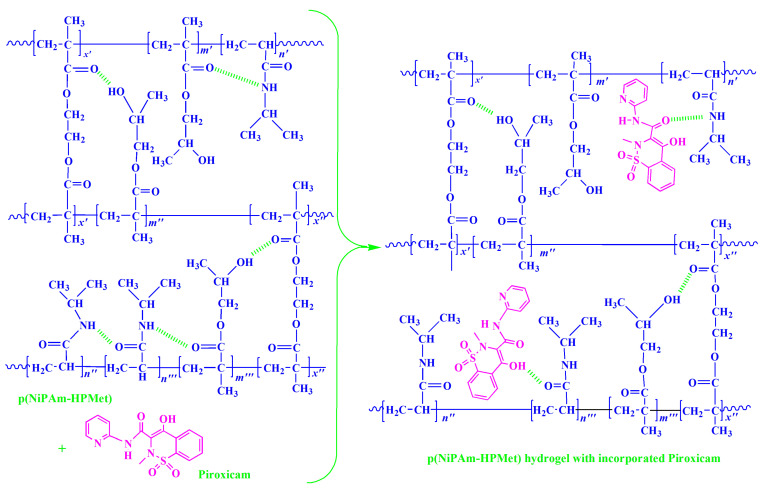
The piroxicam incorporation into the poly(*N*-isopropylacrylamide-*co*-2-hydroxypropyl methacrylate) hydrogels (the potential intramolecular interactions is indicated).

The pore size of the crosslinked matrix in the swelling state provide enough free space for the small piroxicam molecules. In the sample with 3 mol% of crosslinker, the result of piroxicam incorporation into p(NiPAm-HPMet) hydrogel was lower, due to higher crosslinking density. It could be noticed that the obtained efficacy of incorporation occurred because the SO_2_ group did not participate in the construction of hydrogen interactions, but only the secondary -OH group and the carbonyl group from piroxicam did. A schematic presentation of the potential incorporation process with possible intermolecular interactions and the formation of non-covalent hydrogen bonds between side functional groups from hydrogel and piroxicam is given in Figure 4.

### 2.7. SEM Analysis of the Copolymer with Incorporated Piroxicam

**Figure 5:**
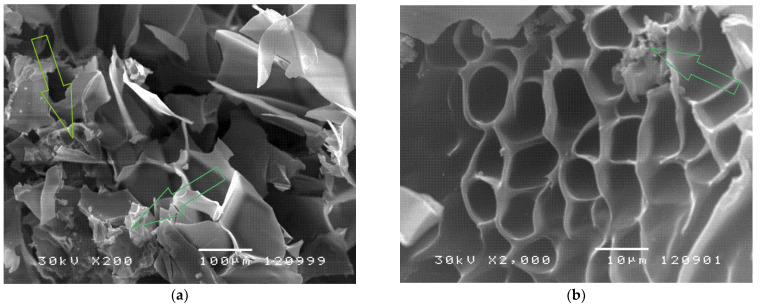
SEM micrographs of poly(*N*-isopropylacrylamide-*co*-2-hydroxypropyl methacrylate) with incorporated piroxicam in the equilibrium swollen state at: (**a**) magnification 200×, scale bar 100 μm; (**b**) magnification 2000×, scale bar 10 μm.

The morphology of poly(*N*-isopropylacrylamide-*co*-2-hydroxypropyl methacrylate) hydrogels swollen to the equilibrium state in the piroxicam solution, and its incorporation into the polymer network, can be seen in Figure 5. The morphology and analysis of p(NiPAM-HPMet) copolymer in the xerogel state, and lyophilized samples swollen to the equilibrium, were presented in the previous study of authors [35] and applied for this analysis.

The topology of poly(*N*-isopropylacrylamide-*co*-2-hydroxypropyl methacrylate) hydrogels with incorporated piroxicam indicates an inhomogeneous distribution of piroxicam within the hydrogel matrix. Certain zones have a preserved structure similar to an empty hydrogel [35]. On the other hand, there are areas that are rich in piroxicam crystals which remained on the three-dimensional surface, which are the first to leave the polymer network upon the release of piroxicam. Otherwise, the piroxicam crystals used in this research are comparableto the pure crystals of piroxicam presented in the study of Cavallari et al. [18].

Obtained results of piroxicam incorporation efficiency, analyses of FTIR spectra and SEM micrographs indicate that piroxicam was satisfactorily incorporated into the pore of synthesized p(NiPAm-HPMet) copolymers.

### 2.8. In Vitro Thermoresponsive Piroxicam Release from p(NiPAm-HPMet) Hydrogels

**Figure 6:**
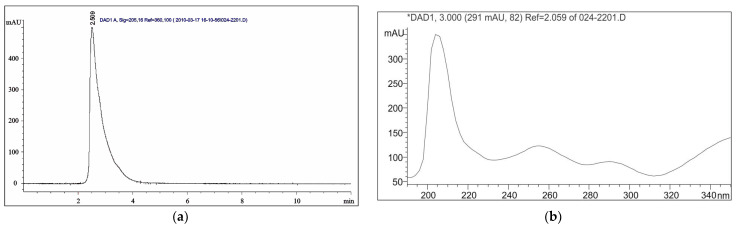
(**a**) HPLC chromatogram of piroxicam, (**b**) UV spectrum of piroxicam from DAD detector.

The released amount of piroxicam from p(NiPAm-HPMet) hydrogels was calculated using Equation (15), valid for the linear part of the calibration curve. The peak on the HPLC chromatogram coming from piroxicam is presented in Figure 6a, and it appears at a retention time of R_t_ = 2.5 min for defined HPLC conditions.

In order to characterize the piroxicam structure, a UV spectrum was recorded, and it showed three absorption maximums (Figure 6b). The first, most intense band at λ_max_ = 205 nm originates from the allowed n→σ* transition of the amide group of heterocyclic benzothiazine. The second, weaker, band at λ_max_ = 255 nm originates from the forbidden π→π* transition -NH-C(=O)^−^. The third, weakest, band at λ_max_ = 290 nm originates from the forbidden n→π* transition of pyridine. According to the data taken from the literature, the neutral form of piroxicam shows a π→π* transition, and the anionic form shows a n→π* transition [5].

**Figure 7:**
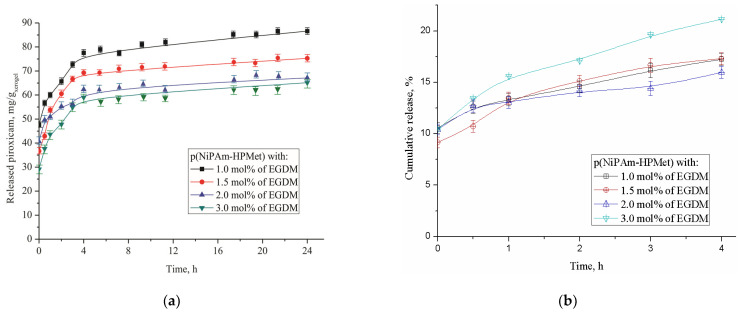
In vitro cumulative release of piroxicam from series of p(NiPAm-HPMet) hydrogels at 37 °C and pH 7.4: (**a**) in mg/g_xerocel_ during 24 h; (**b**) in percentages during first 4 h. Error bars represent the standard deviation of the means of three replicates.

In vitro cumulative release of piroxicam from the synthesized p(NiPAm-HPMet) samples at 37 °C in fluid with pH 7.4 are shown in Figure 7.

The content of released piroxicam during the first 24 h from the poly(*N*-isopropylacrylamide-*co*-2-hydroxypropyl methacrylate) hydrogel sample with the lowest crosslinking density (with 1 mol% of EGDM) shows the highest amount (86.62 mg/g_xerogel_, i.e., 19.24% of the incorporated piroxicam amount). The smallest amount of piroxicam (65.13 mg/g_xerogel_, or 22.13%) was released from the sample with the highest content of EGDM. The obtained results are in agreement with the analyzed swelling behavior, and depend on the hydrogel crosslinking density. It indicates the possibility that thermosensitive p(NiPAm-HPMet) hydrogel could be applied as a drug carrier system with a controlled/modulated release of piroxicam. During the first day, about 77–80% of piroxicam remained within the hydrogels matrix, and this quantity could be suitable for extended release. It is interesting to note that during the first 4 h, about 90% was released, and during the next 20 h, about 10% of the piroxicam was released from the total amount released during 24 h (Figure 7b and Table 9). The relatively small amount of piroxicam released from the hydrogels during one day provides the possibility of continuous release of the remaining drug (about 90%) over a longer time period (probably about 10 days), which could be suitable for topical administration. Design of the new drug carrier could be especially useful for prolonged piroxicam release. Specifically, the chosen crosslinker amount in the planed p(NiPAm-HPMet) hydrogel synthesis determined the desired density of the free spaces in the polymer matrix, which affect the piroxicam incorporation efficacy, and, after that, the diffusion of its molecules through the porous matrix at a temperature above LCST. Thermosensitiveness of synthesized p(NiPAm-HPMet) copolymers was dominant in the piroxicam incorporation at the lower temperature of 10 °C (below LCST), as well as in the aggregation process and its releasing at the higher temperature of 37 °C (above LCST and VPTT), when the hydrogels contract and squeeze fluid with piroxicam. A temperature of 10 °C was chosen as optimal for the incorporation of low soluble piroxicam into swollen hydrogels. At a temperature above the LCST, hydrophobic interactions dominated and caused the breaking of intermolecular hydrogen bonds with piroxicam and p(NiPAm-HPMet), when the drug was released from the contracted hydrogel.

The kinetic parameters of diffusion (*n*, *k*, and *D*) of the piroxicam release mechanism from p(NiPAm-HPMet) copolymers were estimated by fitting the results of the tested delivery using Equations (5)–(8) and are presented in Table 9.

Kinetic parameters for the released piroxicam from the p(NiPAm-HPMet) copolymers, at human body physiological conditions (37 °C and pH 7.4) show that the fluid transport mechanism corresponds to Fickian, i.e., “Less Fickian” diffusion. This mechanism was characterized by penetration of fluid, which is notably slower in relation to the relaxation of polymer chains and is controlled by the process of diffusion (Table 9).

Diffusion coefficient values of the analyzed series of p(NiPAm-HPMet) hydrogels were similar, with little differences between samples. Interestingly, the hydrogel with higher crosslinking density (3 mol%) and the smallest efficacy of incorporation indicate a slightly higher value of the diffusion coefficient, D, and also, a slightly higher value of the piroxicam release rate, with 22.97% of released drug. The reason for this behavior is probably the existence of “microcavities” in the polymer matrix which have a specific effect in the diffusion process [54].

Analysis of the in vitro thermosensitive piroxicam delivery indicates that they would be appropriate for releasing a drug for a prolonged period of time, especially more than one day, in slightly alkaline fluid (pH 7.4), e.g., for topical administration. This topical usage includes applying the drug to the skin to cause local effects while avoiding systemic effects. Topical administration of NSAIDs was recommended by the American College of Rheumatology for knee osteoarthritis [55]. In a recently published study [30], the amount of released piroxicam during 6 h from 0.3% piroxicam-loaded water/oil microemulsion was less than that from 0.5% piroxicam marketed gel. In most of the formulations from the polymeric reservoir based on chitosan and hydroxy propyl methyl cellulose, a hydrodynamically balanced for sustained-release, for stomach-specific delivery of piroxicam by using gastroretention technology, was explained by Fickian diffusion, which corresponds to polymer relaxation when they come in contact with dissolution fluid [26].

According to the obtained results in this work, it was shown that a drug delivery system of thermosensitive p(NiPAm-HPMet) hydrogels with piroxicam would be of interest for further testing for modulated, topical piroxicam administration.

## 3. Conclusions

The achieved conversion of the NiPAm and HPMet as comonomers and EGDM as the crosslinker into the p(NiPAm-HPMet) copolymers was satisfied, and residual amounts of reactants in all samples were within tolerance limits, which is requiredfor their biomedical applications. The phase transition identification of p(NiPAm-HPMet) copolymersusing DSC method showed weak intensity of more than one glass transition of amorphous regions, and two sharp peaks from the melting of crystalline regions. It confirmed that p(NiPAm-HPMet) hydrogels are thermally stable up to 150 °C, which allows for their sterilization, as a very important detail for biomedical applications. The swelling reversibility of p(NiPAm-HPMet) copolymers was confirmed in three cycles. The fluid transport mechanism into the hydrogel samples at 10 °C and 40 °C was calculated, and it was subjected to Fick’s law at 40 °C. The swelling process of hydrogels follows a second-order reaction. The structural and morphological changes in the p(NiPAm-HPMet) copolymer with incorporated piroxicam were confirmed by using FTIR and SEM methods. Piroxicam was satisfactorily incorporated, especially in the sample with 1 mol% of EGDM. The in vitro thermosensitive piroxicam release from the p(NiPAm-HPMet) hydrogels showed that it could be attractive for an additional study for controlled/modulated piroxicam delivery over a very long time.

## 4. Materials and Methods

### 4.1. Reagents

*N*-Isopropylacrylamide 99%, 2,2′-azobis(2-methylpropionitrile) 98% and 2-hydroxypropyl methacrylate 96.5% (Acros Organics, Morris Plains, NJ, USA); ethylene glycol dimethacylate 97% and methanol Chromasolv 99.9% HPLC grade (Fluka Chemical Corp., Buchs, Switzerland); piroxicam 99.67% (Megafine Pharma (P) Ltd., Maharashtra, India); methanol, p.a. (Unichem, Belgrade, Serbia); potassium bromide for IR spectroscopy (KBr) ≤100%, (Merck KGaA, Darmstadt, Germany); acetone (Centrohem, Belgrade, Serbia).

### 4.2. Thermosensitive Hydrogels Synthesis

Three-dimensional hydrogels based on monomer *N*-isopropylacrylamide were synthesized using the free radical polymerization method with comonomer 2-hydroxypropyl methacrylate (15 mol% relative to the NiPAm monomer amount) [35]. The ethylene glycol dimethacylate was added for crosslinking linear chains (0.5, 1.0, 1.5, 2.0 and 3.0 mol% relative to the total mass of monomers in the reaction mixtures). The polymerization process was initiated using 2.7 mol% of 2,2′-azobis(2-methylpropionitrile), relative to the amount of NiPAm monomer, and all reactants were diluted in acetone. The reaction samples after homogenization were injected into glass ampoules. All sealed samples were made warm in the temperature regime: 100 min at 75 °C, 70 min at 80 °C, and 20 min at 85 °C. The polymerized copolymers were sliced into cylinders (diameter 5mm, thickness 2mm). In order to the remove of residual reactants, sliced samples were submerged by methanol for the time of 2 days, after which they were submerged for a day into solutions of methanol:distilled water in 80:20, 60:40, 40:60, 20:80 and 0:100% with stirring. After purification, swollen cylinders were dried by evaporation of solvents at 40 °C.

### 4.3. Residual Reactants Analysis

The residual amounts of reactans: EGDM as crosslinker and monomers NiPAm and HPMet in the samples after polymerization of poly(*N*-isopropylacrylamide-*co*-2-hydroxy propyl methacrylate) hydrogels were extracted in methanol during 48 h with stirring. All extracted and decanted methanol samples were filtered using a 0.45 μm cellulose membrane filter. The apparatus HPLC Agilent 1100 Series with a diode-array detector (DAD) (Agilent Technologies, Santa Clara, CA, USA) was used for analysis. The detector was adjustedto a 220 nm wavelength. The ZORBAX Eclipse XDB-C18 column (250 mm × 4.6 mm, 5 μm, Agilent Technologies, Santa Clara, CA, USA) was tuned at 25 °C. The flow rate of methanol, as a mobile phase was 1 cm^3^/min. The volume of the injected sample was 10 μL. The quantification of the residual amounts of reactans was performed using a method previously developed by Ilić-Stojanović and colleagues [39,56]. Constructed calibration curves, HPLC chromatograms and Equations (1)–(3), for peak areas depending on concentrations of NiPAm, HPMet, and EGDM, respectively, described in the previous studies, [39,56], were applied for calculation:

(1) $$c_{\mathrm{NiPAm}\;}=\frac{A_{\mathrm{NiPAm}\;}-113.0}{26652.8}$$

(2) $$c_{\mathrm{HPMet}}=\frac{A_{\mathrm{HPMet}}-453.3}{10164.7}$$

(3) $$c_{\mathrm{EGDM}}=\frac{A_{\mathrm{EGDM}}-340.8\;}{64303.3}$$

where *c*_NiPAm,_ *c*_HPMet,_ *c*_EGDM_ are concentration (mg/cm^3^), and *A*_NiPam_, *A*_HPMet_, *A*_EGDM_ are pick area (mAU·s) of reactants NiPAm, HPMet and EGDM, respectively. 

### 4.4. Swelling Study

The process of the p(NiPAm-HPMet) hydrogels swelling was supervised using the gravimetric method at 10 °C and 40 °C, analog as in the previously published study [35]. Polymerized xerogels were first measured and then submersed in fluid with pH 7.4. At a determined time, the samples were pulled out, the masses were weighed and then taken back in the vial while a constant mass was reached. The equilibrium swelling ratio, α_∞_, was calculated by Equation (4):

(4) $$\alpha_{\infty }=\frac{m_{\infty }-m_{o}}{m_{o}}$$

*m*_o_—mass of xerogel, *m_t_*—the swollen hydrogel mass at the time *t*, *m*_∞_—mass of the swollen hydrogel at equilibrium.

#### 4.4.1. Kinetic

For the purpose of analyzing the nature of the process of fluid diffusion into the p(NiPAm-HPMet) copolymers during swelling and the piroxicam delivery processes, Fick’s Equation (5) was used to fit and count the experimental data [42,43].

(5) $$\frac{M_{t}}{M_{\infty }}=kt^{n}$$

*M_t_*—the absorbed fluid amount at the time *t*, *M_t_*/*M*_∞_—the fractional sorption, *M*_∞_—the absorbed fluid amount at the equilibrium, *k*—the kinetic constant (min^1/n^), *n*—the diffusion exponent. If *n* < 0.5, the swelling mechanism is controlled by the Fickian diffusion, whereas a value of 0.5 < *n* < 1 indicates an anomalous non-Fickian type diffusion and contributes to the water-sorption process. If *n* = 0.5, diffusion degree is much lower than the polymer chain relaxation degree (Case I), whereas *n* = 1 indicates the fluid diffusion process is much faster than the relaxation of polymer system chains (Type II, Case II). When value of *n* > 1, the polymer chain relaxation controls swelling, and is called Type III (Case III, Super Case II).

By taking the logarithm of the Equation (5), the Equation (6) is obtained:

(6) $$\ln F=\ln(M_{t}/M_{\infty })=\ln k+n\ln t$$

The exponents *n* and kinetic constant *k* values were calculated from the slope and intercept of the linear relation between ln *F* and ln *t*. The diffusion coefficient *D* was calculated for 60% of the swelling (in initial phase) from Equation (7) [44,45,46]:

(7) $$\frac{M_{t}}{M_{\infty }}=\left(\frac{4}{\pi^{0.5}}\right){\left(\frac{D_{t}}{l^{2}}\right)}^{0.5}$$

*l*—the thickness of the dried sample, cm, *D*—the diffusion coefficient, cm^2^/min. By taking the logarithm of the Equation (7), Equation (8) is obtained (the linear relationship between ln (*M_t_/M_e_*) and ln *t*):

(8) ln(MtMe)=ln(4D12π12l)+12lnt

The diffusion coefficient can be determined from the intercept of the linear relation between ln(*M_t_*/*M_e_*) and ln*t*; (*t*—the time for which the xerogel absorbs half of the total fluid amount).

#### 4.4.2. The Order of a Swelling Reaction

Based on the swelling kinetics results, the order of a swelling process was calculated for the p(NiPAm-HPMet) hydrogels. The normalized swelling degree, *α_t_*, is determined using Equation (9):

(9) $$\alpha_{t}=\frac{w_{t}}{m_{0}}$$

*m*_0_—the xerogel mass, *w_t_*—the mass of absorbed liquid at period of time *t*. The normalized equilibrium swelling degree, *α*_e_, is calculated using Equation (10):

(10) $$\alpha_{e}=\frac{w_{e}}{m_{0}}$$

*m*_0_ is the xerogel mass and *w*_e_ is the mass of liquid absorbed by the gel in the equilibrium state. Using the calculated values for *α_t_* and *α*_e_, the reaction order of the hydrogel swelling process was determined. If the swelling kinetics follows a first-order reaction, the results can be calculated by Equation (11):

(11) $$\frac{\alpha_{t}}{\alpha_{e}}=1-e^{-Kt}$$

*K*—the reaction rate constant. The required condition is that the logarithmic dependence 12 be linear, with a slope of −*K*, i.e.:

(12) $$\ln\frac{\alpha_{e}-\alpha_{t}}{\alpha_{e}}=-Kt$$

It is supposed that the process of swelling follows a first-order reaction, if this dependence 12 is linear. If this requirement is not met, it is than checked whether the swelling process follows a second-order reaction or not. The kinetics of the swelling process, which follows a second-order reaction, are described by Equation (13):

(13) $$\frac{t}{\alpha_{t}}=\frac{1}{K\alpha_{e}^{2}}+\frac{t}{\alpha_{e}}$$

The requirement is that the dependence 13 be linear. The values of the normalized equilibrium degree of swelling *α*_e_, the rate constant, *K*, and the linear correlation coefficient, R^2^, were determined from the experimental results using logarithmic Equations (12) and (13).

### 4.5. Incorporation of Piroxicam into the Thermosensitive Hydrogels

Due to its very low solubility, piroxicam standard substance was dissolved in acidified ethanol (1 × 10^−3^ mol/dm^3^ hydrochloric acid, p.a. 36.5%, HCl) at an elevated temperature of 40 °C. For their loading into the thermosensitive p(NiPAm-HPMet) hydrogels, analyzed samples in xerogel state were swollen in the prepared piroxicam solution, 40 mg/cm^3^ during 48 h at 10 °C. Piroxicam incorporation efficiency (*η*) was counted by applying Equation (14):

(14) $$\eta (\%)=\frac{L_{g}}{L_{u}}\times 100$$

*L*_g_—the mass of piroxicam incorporated in the hydrogel sample (mg/g_xerogel_) and *L*_u_—the maximum of piroxicam available mass for incorporation (mg/g_xerogel_).

### 4.6. In Vitro Piroxicam Release from Thermosensitive Hydrogels

The swollen p(NiPAm-HPMet) copolymers with loaded piroxicam were overflowed with 7 cm^3^ of fluids at pH 7.4 and 37 °C, and piroxicam release was supervised during 24 h. Portions of 100 µL of the fluid with released piroxicam were taken in definite intervals of time, diluted with methanol and filtered using a 0.45 μm cellulose membrane filter for analysis. The analysis was carried out on the HPLC Agilent 1100 Series device (Agilent Technologies, Santa Clara, CA, USA) under the following conditions: diode-array detector, DAD 1200 Series, column Zorbax Eclipse XDB-C18 250 × 4.6 mm, 5 µm (Merck KGaA, Darmstadt, Germany),the eluent was methanol for HPLC, 99.9% gradient grade, the eluent flow 1 cm^3^/min, sample injected volumes 10 μL and detector wavelength 205 nm for the piroxicam standard substance. For the calibration curve construction, a series of known concentrations of piroxicam standard solutions were prepared, filtered using a 0.45 μm cellulose membrane filter for analysis. The obtained chromatograms were analyzed by using Agilent ChemStation software. The linear part of calibration curve in the range 0.005–0.250 mg/cm^3^ (correlation coefficient *R*^2^=0.9987), and the released amount of piroxicam from p(NiPAm-HPMet) hydrogels, was determined based on Equation (15):

(15) $$c_{\mathrm{piroxicam}\;}=\frac{A-681}{58507.7}$$

where *c*_piroxicam_ is the piroxicam content (mg/cm^3^) and *A* is the peak area (mAU∙s).

### 4.7. Characterization

#### 4.7.1. DSC Method

Thermal properties of the p(NiPAm-HPMet) hydrogel samples were investigated by differential scanning calorimetry (DSC) by using a DSC Q20 apparatus (TA instruments). Aluminum pans containing 3–5 mg of xerogels were hermetically sealed and heated from 30 °C to 180 °C, with the heating rate of 10 °C min^−1^. Standard calibration was performed by using indium. The sensitivity of the instrument is 10 m V cm^−1^.

#### 4.7.2. FTIR Method

Pure p(NiPAm-HPMet) sample in xerogel state, piroxicam, and p(NiPAm-HPMet) xerogel with loaded piroxicam were shredded in the Amalgamator (WIG-L-Bug, Dentsply RINN, a Division of Dentsply International Inc., Smile Way York, USA). Powder samples (0.9 mg) with the potassium bromide (about 150 mg) were vacuumed and put under pressure of about 200 MPa to form transparent tablets. FTIR spectra of all samples were recorded on a FTIR spectrophotometer Bomem Hartmann & Braun MB-series (Hartmann & Braun, Baptiste, CA, USA) in the area of wavelength from 4000 to 400 cm^−1^, at the resolution of 2 cm^−1^ and with 16 scans. FTIR spectra were analyzed by using Win-Bomem Easy software for analysis.

#### 4.7.3. Lyophilization Process

Lyophilization process of obtained p(NiPAm-HPMet) hydrogels in equilibrium swollen state, pure and with incorporated piroxicam, was performed on the device LH Leybold, Lyovac GT2 (Frenkendorf, Switzerland). Firstly, the hydrogels swollen in equilibrium state were cooledat −40 °C during 24 h. Secondly, the fluid was removed by sublimation at −30 °C and 0.05 kPa during 18 h. After that, in the isothermal desorption phase at 0.05 kPa, the hydrogels were heated at 20 °C during 6 h. Obtained lyophilized hydrogels were packed under vacuum condition and stored at 5–7 °C.

#### 4.7.4. Scanning Electron Microscope (SEM) Method

Analyzed lyophilized samples of p(NiPAm-HPMet) hydrogels in equilibrium swollen state, before and after piroxicam loading, were metalized first with a gold/palladium (85/15) alloy under vacuum using JEOL Fine Coat JFC 1100E Ion Sputter (JEOL Ltd., Tokyo, Japan), and then p(NiPAm-HPMet) hydrogels were scanned on a JEOL Scanning Electron Microscope JSM-5300 (JEOL Ltd., Tokyo, Japan).

### 4.8. Statistical Analysis

Analysis of variance (ANOVA) was applied to determine the significance (*p* ≤ 0.05) of the data obtained in the experiments of swelling process, and in vitro piroxicam delivery. All results were calculated to be within the 95% confidence level for reproducibility.

## 5. Patents

Registered patent RS 53220 B: Ilić-Stojanović, S.; Nikolić, Lj.; Nikolić, V.; Petrović, S.D.; Stanković, M. Process for synthesis of thermosensitive hydrogels and pharmaceutical applications, The Intellectual Property Office of the Republic of Serbia, https://worldwide.espacenet.com/patent/search/family/046025219/publication/RS53220B?q=RS53220B, accessed on 1 May 2022.

## Figures and Tables

**Table 1:** Amounts of residual reactants NiPAm, HPMet and EGDM in relation to the initial amount in the reaction mixture, in mass%.

p(NiPAm-HPMet) with mol% of Crosslinker EGDM	NiPAm, %	HPMet, %	EGDM, %
1.0	0.36	0.27	0.66
1.5	0.45	0.32	0.62
2.0	0.46	0.47	0.45
3.0	0.47	0.38	0.31

**Table 2:** The glass transition temperatures (*T*_g_), melting temperatures (*T*_m_) and the melting enthalpy (ΔH_m_) values for the analyzed p(NiPAm-HPMet) xerogels.

p(NiPAm-HPMet) with mol% of Crosslinker EGDM	*T*_g1_, ( °C)	*T*_g2_, ( °C)	*T*_g3_, ( °C)	*T*_m1_, ( °C)	*T*_m2_, ( °C)	ΔH_m_, (J∙g^−1^)
1.0	71.26	-	129.81	152.36	156.49	6.48
2.0	63.40	81.84	134.67	150.93	155.27	3.55
3.0	62.88	81.05	135.95	150.72	153.14	2.94

**Table 3:** The equilibrium swelling ratio (*α*_e_) and kinetic parameters (*n*, *k* and *D*) of the fluid diffusion into p(NiPAm-HPMet) hydrogels at 10 °C and pH 7.4.

p(NiPAm-HPMet) with mol% of EGDM	*α*_e_	*n*	*k* × 10^2^ (min^–1/2^)	R^2^	*D* (cm^2^/min)
1.0	26.70	0.395	6.323	0.997	1.041 × 10^−5^
1.5	18.04	0.575	2.987	0.996	8.872 × 10^−7^
2.0	15.10	0.500	2.720	0.989	1.414 × 10^−6^
3.0	12.03	0.435	2.472	0.996	0.186 × 10^−6^

**Table 4:** The equilibrium swelling ratio (*α*_e_) and kinetic parameters (*n*, *k* and *D*) of the fluid diffusion into p(NiPAm-HPMet) hydrogels at 40 °C.

p(NiPAm-HPMet) with mol% of EGDM	*α*_e_	*n*	*k* × 10^2^ (min^–1/2^)	R^2^	*D* × 10^4^ (cm^2^/min)
1.0	3.94	0.434	0.095	0.999	2.979
1.5	2.61	0.433	0.747	0.995	1.206
2.0	2.04	0.290	0.142	0.997	2.016
3.0	1.53	0.379	0.111	0.983	2.594

**Table 5:** Values of the experimentally obtained and calculated normalized equilibrium swelling degree and kinetic parameters of the first and second order reaction at 10 °C.

p(NiPAm-HPMet) with mol% of EGDM	*α*_e_ (exp)	*α*_e_ (I order)	*K*·10^3^, min^−1^	R^2^	*α*_e_ (II order)	*K*·10^3^, min^−1^	R^2^
1.0	26.70	28.621	4.54	0.968	27.504	0.344	0.999
1.5	18.04	19.628	3.87	0.974	18.693	1.014	0.999
2.0	15.10	16.002	2.71	0.988	15.344	3.118	0.999
3.0	12.03	12.914	3.54	0.990	12.413	2.247	0.999

**Table 6:** Values of the experimentally obtained and calculated normalized equilibrium swelling degree and kinetic parameters of the first and second order reaction at 40 °C.

p(NiPAm-HPMet) with mol% of EGDM	*α*_e_ (exp)	*α*_e_ (I order)	*K*·10^3^, min^−1^	R^2^	*α*_e_ (II order)	*K*·10^3^, min^−1^	R^2^
1.0	3.94	4.672	7.58	0.994	4.223	6.081	0.999
1.5	2.61	3.254	7.24	0.989	2.978	5.173	0.999
2.0	2.04	2.904	6.47	0.988	2.561	5.417	0.999
3.0	1.53	1.771	6.89	0.991	1.65	4.171	0.998

**Table 7:** Positions of characteristic absorption band maximums for p(NiPAm-HPMet), piroxicam p(NiPAm-HPMet), with incorporated piroxicam and value of wavelengths shifts after drug incorporation.

Wavenumber of Functional Group, cm^−1^			Functional Group	Shifts in Relation to the FTIR Spectra, cm^−1^	
p(NiPAm-HPMet)	Piroxicam	p(NiPAm-HPMet) with Piroxicam		p(NiPAm-HPMet)	Piroxicam
3438		3449	ν(OH)	+11	
3319			ν(NH)	−	
	3393	3393	ν(NH)		0
	3338	3346	ν(OH)		+8
	3066		ν(C-H) pyridine		
2973	2934	2973	ν_as_(CH_3_)	0	
2933		2928	ν_as_(CH_2_)	−5	−6
2876		2857	ν_s_(CH_3_)	+19	
1728	1742	1731	ν_s_ (C=O)	+3	−11
1650	1630	1636	ν_s_(C=O) amide I	−14	+6
	1560, 1474	1563, 1474	ν(C=C) Ar		+3, 0
	1576	1577	ν(C-N) pyridine		+1
1544	1531	1531	δ(NH) amide II	−13	0
1460	1436	1437	δ(OH)	−23	+1
1378			δ(CN)- isopropyl	−10	
	1351	1351	ν_as_(SO_2_)		0
	1300	1300	ν(C-N) amide III		0
	1182	1182	ν_s_(SO_2_)		0
1173			ν_as_(C-O)		
1131	1155	1156	ν_s_(C-O)	+25	+1
925	939	939	γ(OH)	+14	0
837	876, 831, 775	876, 831, 775	δ(C-H) Ar	0, 0, 0	0
	732, 691		γ(C-H)		
674	692	669	γ(OH)	−5	-

**Table 8:** The Mass of incorporated piroxicam (*L*_g_) into p(NiPAm-HPMet) samples and piroxicam incorporation efficiency (*η*_piroxicam_).

p(NiPAm-HPMet) with mol% of Crosslinker EGDM	*L*_g_ Piroxicam (mg/g_xerogel_)	*η*_piroxicam_ (%)
1.0	331.35	66.34
1.5	273.39	54.79
2.0	234.45	47.08
3.0	191.83	38.45

**Table 9:** Kinetic parameters of diffusion (*n*, *k* and *D*) for piroxicam released from p(NiPAm-HPMet) copolymers at 37 °C and pH 7.4.

p(NiPAm-HPMet) with EGDM	mg_piroxicam_/g_xerogel_	*n*	*k* (min^−1/2^)	R^2^	*D* × 10^3^ (cm^2^/min)	%, First 24 h	%, First 4 h
1.0 mol%	86.616	0.062	0.646	0.979	3.28	19.24	17.23
1.5 mol%	75.246	0.099	0.543	0.988	3.32	18.81	17.31
2.0 mol%	67.134	0.061	0.719	0.989	4.07	17.28	15.97
3.0 mol%	65.133	0.054	0.948	0.986	4.14	22.97	21.13

## Data Availability

Not applicable.
